# Toscana, West Nile, Usutu and tick-borne encephalitis viruses: external quality assessment for molecular detection of emerging neurotropic viruses in Europe, 2017

**DOI:** 10.2807/1560-7917.ES.2019.24.50.1900051

**Published:** 2019-12-12

**Authors:** Chantal Reusken, Cecile Baronti, Ramona Mögling, Anna Papa, Katrin Leitmeyer, Remi N Charrel

**Affiliations:** 1 Department of Viroscience, Erasmus University Medical Centre, Rotterdam, the Netherlands; 2 Centre for Infectious Disease Control, National Institute for Public Health and the Environment (RIVM), Bilthoven, the Netherlands; 3 Unite des Virus Emergents (UVE: Aix Marseille Univ, IRD 190, INSERM 1207, IHU Mediterranee Infection), Marseille, France; 4 Department of Microbiology, Medical School, Aristotle University of Thessaloniki, Thessaloniki, Greece; 5 European Centre for Disease Prevention and Control (ECDC), Solna, Sweden

**Keywords:** arbovirus, emerging, meningitis, central nervous system, encephalitis, diagnostic capacity, external quality assessment, Toscana virus, TOSV, Usutu, USUV, West Nile, WNV, tick-borne encephalitis, TBE

## Abstract

**Background:**

Neurotropic arboviruses are increasingly recognised as causative agents of neurological disease in Europe but underdiagnosis is still suspected. Capability for accurate diagnosis is a prerequisite for adequate clinical and public health response.

**Aim:**

To improve diagnostic capability in EVD-LabNet laboratories, we organised an external quality assessment (EQA) focusing on molecular detection of Toscana (TOSV), Usutu (USUV), West Nile (WNV) and tick-borne encephalitis viruses (TBEV).

**Methods:**

Sixty-nine laboratories were invited. The EQA panel included two WNV RNA-positive samples (lineages 1 and 2), two TOSV RNA-positive samples (lineages A and B), one TBEV RNA-positive sample (Western subtype), one USUV RNA-positive sample and four negative samples. The EQA focused on overall capability rather than sensitivity of the used techniques. Only detection of one, clinically relevant, concentration per virus species and lineage was assessed.

**Results:**

The final EQA analysis included 51 laboratories from 35 countries; 44 of these laboratories were from 28 of 31 countries in the European Union/European Economic Area (EU/EEA). USUV diagnostic capability was lowest (28 laboratories in 18 countries), WNV detection capacity was highest (48 laboratories in 32 countries). Twenty-five laboratories were able to test the whole EQA panel, of which only 11 provided completely correct results. The highest scores were observed for WNV and TOSV (92%), followed by TBEV (86%) and USUV (75%).

**Conclusion:**

We observed wide variety in extraction methods and RT-PCR tests, showing a profound absence of standardisation across European laboratories. Overall, the results were not satisfactory; capacity and capability need to be improved in 40 laboratories.

## Background

The aetiology of neuro-invasive viral infections remains undetermined in more than 50% of cases [1]. Several viruses can cause infections of the central nervous system (CNS) while, regardless of the causative aetiology, clinical manifestations are often similar, making a confirmed diagnosis dependant on laboratory testing [2]. Neurotropic arboviruses are increasingly recognised as causative agents of neurological disease in Europe but underdiagnosis is still suspected [3]. Confirmed involvement of arboviruses is important for risk communication and risk management strategies, the latter including activities like local vector control, blood safety measures and vaccination campaigns. Four neurotropic arboviruses are emerging and have become endemic in large parts of Europe: Toscana virus (TOSV), Usutu virus (USUV), West Nile virus (WNV) and tick-borne encephalitis virus (TBEV).

The TOSV (genus *Phlebovirus,* family *Phenuiviridae*) is transmitted by sandflies of the genus *Phlebotomus* and circulates in Mediterranean countries where it can cause febrile illness and neuroinvasive infections. At least 250 million people are exposed in Europe and neighbouring countries around the Mediterranean basin that are frequently visited by travellers for occupational or leisure purposes [4–7]. In France, Spain and Italy, TOSV is among the three most common agents causing aseptic meningitis and encephalitis, together with enteroviruses and herpesviruses (herpes simplex and varicella–zoster viruses) [8]. Viraemia is short-lived (typically 5 days, range: 2–7) and diagnosis is done either by detecting viral RNA in cerebrospinal fluid (CSF) or serum at the acute stage of infection or by detecting IgM in an early serum sample [8]. The currently known circulation of three genetic lineages may be indicative of a wide genetic diversity of this viral species and thus molecular assays are needed to detect genetic variants [8]*.*

WNV (genus *Flavivirus*, family *Flaviviridae*) is transmitted by *Culex* spp. mosquitoes. WNV can cause febrile illness with or without neurological manifestations. During the last decade, WNV activity in Europe has shown a profile similar to that observed in North America, with substantial activity reported every year and with recurring major outbreaks [9,10]. Major recent activity in the eastern Mediterranean region is also a matter of concern for Europe [11].

Lineages 1 and 2 have been identified in human WNV cases in Europe [12]. Severe cases are more frequent in elderly and immunocompromised patients. In the acute stage of disease, WNV RNA can be detected in CSF. WNV viraemia is typically short-lived, but viral RNA can be detected for longer periods in some specimens such as urine and whole blood, and also in fatal cases or immunocompromised patients. The high degree of cross-reactivity with other flaviviruses in serology is problematic. Although a combination of serology and PCR is desirable, the detection of WNV RNA alone is an important means of undisputable confirmation of acute infection [13].

USUV (genus *Flavivirus*, family *Flaviviridae*) was first isolated in Africa in 1959 [13]. It is a *Culex*-transmitted flavivirus closely related to WNV [13]. The earliest human cases (presenting as neuro-invasive disease) were recorded in 2009 in Italy in two immunocompromised patients having received blood products [13]. Since then evidence of zoonotic transmission accompanied by neurological disease of USUV is accumulating while population studies show the occurrence of asymptomatic infections [13]. Nothing is known about the length of USUV viraemia and the kinetics of antibody production in humans [13]. Based on its close relatedness to WNV, viraemia is expected to be short and low level [13]. At the acute stage of neuroinvasive infection the virus is expected to be detectable by RT-PCR in CSF. A high degree of cross-reactivity with other flaviviruses is seen in serology. For this reason, molecular detection is the preferred method for confirmatory laboratory diagnosis.

TBEV (genus *Flavivirus*, family *Flaviviridae*) is a tick-borne flavivirus; the incidence of TBEV infection in humans and its geographical distribution have increased in Europe [14]. Three subtypes are recognised, of which the Western subtype is endemic in northern, central and eastern Europe. The clinical spectrum of the disease ranges from mild meningitis to severe meningoencephalitis. The course of TBE is often biphasic. The first acute phase typically has non-specific symptoms. It is followed by an asymptomatic interval that precedes the second phase characterised by neuroinvasive disease. Therefore, the window of molecular detection in serum and CSF is often missed as diagnostics are typically requested during the second phase of illness. Once neurological symptoms are manifest, TBEV RNA is rarely detected in blood and CSF. However, in endemic regions, unexplained febrile illness alone can justify molecular testing. As TBEV cases often do not present with typical symptoms, diagnosis often relies on laboratory documentation. As for WNV, viraemia is typically short-lived and low while serology is hampered by extensive cross-reactivity among flaviviruses [15].

To support molecular diagnostic capacity and capability building for these emerging neurotropic viruses in the European Union(EU)/ European Economic Area (EEA) and EU pre-accession countries, an external quality assessment (EQA) was organised for members of the expert laboratory network EVD-LabNet (https://www.evd-labnet.eu/) funded by the European Centre for Disease Prevention and Control. Here, we present this assessment and the inventory of methods for RT-PCR detection of these viruses that are used in European and national reference laboratories.

## Methods

### EQA scheme organisation

All members of EVD-LabNet (69 laboratories at 1 November 2017) were invited by email to participate through online registration. Fifty-four laboratories from 35 countries registered online.

### Panel composition

The EQA panel consisted of 10 samples with six samples positive for one of four different viral species (plasma samples spiked with viruses), and four negative control samples. The panel included two WNV RNA-positive samples (WNV lineage 1, strain UVE/WNV/2001/FR/DON2001 (ref#001V-02215), 7.2 × 10^4^ RNA copies/0.4 mL [16] and WNV lineage 2, strain B956 source BNI, Hamburg, 4.96 × 10^5^ RNA copies/0.4 mL), two TOSV RNA-positive samples (TOSV lineage A, strain UVE/TOSV/2010/TN/ T152 (ref#001V-02119), 1.57 × 10^5^ RNA copies/0.4 mL [17] and TOSV lineage B, strain UVE/TOSV/2010/FR/4319 (ref#001V-02442), 1.24 × 10^5^ RNA copies/0.4 mL [18]), one TBEV RNA-positive sample (Western subtype, strain UVE/TBEV/1953/CZ/Hypr (ref#001V-EVA134), 5.06 × 10^4^ RNA copies/0.4mL [19]), one USUV RNA-positive sample (USUV, strain *Turdus merula* NL2016 (ref#011V-02153), 6.34 × 10^3^ RNA copies/0.4 mL) [20] and four viral RNA-negative plasma samples. Strains referenced in the European Virus Archive (EVA) can be accessed at https://www.european-virus-archive.com. 

Each sample of the panel was prepared from a batch that consisted of qualified non-therapeutic human plasma obtained from the French blood bank, spiked with virus culture supernatant and heat-inactivated at 60 °C for 1 hour. A total of 70 0.4-mL aliquots were prepared and freeze-dried into glass vials. Proper inactivation was confirmed by the absence of cytopathic effect in Vero cells and by undetectable increase of the viral RNA titre in the supernatant 5 days after inoculation. The viral loads per reconstituted sample were quantified with reference to in-house TOSV-, WNV-, TBEV- and USUV-specific synthetic RNA controls; a fragment (ca 500 bp) tagged at the 5’‑end with the T7 promoter sequence (5’‑TAATACGACT CACTATAGGG‑3’) and containing the virus-specific TaqMan-targeted sequence was amplified by RT-PCR using the Access RT-PCR kit (Promega, Charbonnières-les-Bains). The resulting PCR products were purified and transcribed using the T7 Megashort script kit (Ambion, ThermoFisher Scientific, Bourgoin-Jallieu). The obtained RNA was purified with the MegaClear purification kit (Ambion, Bourgoin-Jallieu). RNA concentration was measured using a NanoDrop 1000 (Thermo Scientific, Bourgoin-Jallieu) and translated into copy numbers. Real-time RT-PCR was performed using the Express One-Step Superscript qRT-PCR Kit, universal (Life technologies, Bourgoin-Jallieu) on a QuantStudio 12K Flex Real-Time PCR System. For each EQA sample, the number of copies contained in 0.4 mL of freeze-dried material in the glass vial was calculated by comparison with a dilution series of T7-generated RNA standard containing 10^2^ to 10^8^ RNA copies.

### Result submission, evaluation and EQA scoring

We provided the Laboratories with a link to an online form to submit their EQA results. Laboratories could indicate for which of the four target viruses they had tested the EQA panel and background information of the diagnostic tests that the laboratory assessed with the EQA. Data were collected and analysed in Microsoft Excel 2011. Fisher's exact test (www.socscistatistics.com/tests/fisher/Default2.aspx) was used to compare the rate of false-negative results obtained with virus-specific real-time assays and with other assays for TOSV, USUV, TBEV and WNV. Fisher's exact test was used because the significance of the deviation from a null hypothesis can be calculated exactly, rather than relying on an approximation that becomes exact in the limit as the sample size grows to infinity, as with the chi-squared test.

## Results

### EQA participation

The final EQA analysis included 51 laboratories form 35 countries: 44 laboratories from 28 EU/EEA countries, four laboratories from four EU pre-accession countries (Albania, North Macedonia, Serbia and Turkey) and three laboratories from three non-EU/EEA countries (Israel, Russia and Switzerland). From the EU/EEA, there was no participation from laboratories in Iceland and France besides the reference laboratory in Marseille that produced the panel. Liechtenstein does not have a reference laboratory participating in EVD-LabNet. From EU pre-accession countries, there was no participation by Bosnia and Herzegovina, Montenegro and Kosovo*, the two latter being not members of EVD-LabNet at the time.

### Nucleic acid extraction methodology

The different techniques used for extraction of nucleic acids are presented in the Table 1. Various Qiagen kits were used by 31 laboratories: 21 used the QIAamp Viral RNA Mini Kit (Qiagen, Hilden) and the remaining 10 laboratories used six different Qiagen kits. The extraction kits from Roche (Meylan) were the second most frequently used brand, with eight laboratories using four different types of Roche kits. Because of the high diversity, it was impossible to include the type of RNA purification in the analysis.

**Table 1:** Nucleic acid extraction methods used in the external quality assessment for molecular detection of emerging neurotropic viruses, Europe (n = 51 laboratories)

Extraction method	Number of laboratories
QIAamp Viral RNA Mini Kit (Qiagen, Hilden)	21
NucliSENSE EasyMag (BioMérieux, Marcy-L'étoile)	4
EZ1 Virus Mini Kit (Qiagen, Hilden)	3
MagNA Pure 96 DNA and Viral NA kit (Roche, Meylan)	3
RNeasy Mini kit (Qiagen, Hilden)	2
QIAamp MinElute Virus Spin Kit (Qiagen, Hilden)	2
MagNa Pure LC total NA kit (Roche, Meylan)	2
MagNa Pure Compact NA isolation kit (Roche, Meylan)	2
iPrep PureLink Virus Kit (Thermo Fisher, Bourgoin-Jallieu)	2
QIAamp DSP Virus (Qiagen, Hilden)	1
Maxwell RSC Viral Total NA Purification Kit (Promega, Charbonnières-les-Bains)	1
QIAxtractor VX (Qiagen, Hilden)	1
QIAamp RNA Blood Mini Kit (Qiagen, Hilden)	1
MagCore Viral NA extraction kit (RBCBioscience, New Taipei City)	1
TriPure isolation reagent (Sigma-Aldrich, Saint-Louis)	1
High Pure Viral RNA kit (Roche, Meylan)	1
NucleoSpin RNA Virus (Macherey-Nagel, Düren)	1
MagDea NA extraction kit for magLead (PSS-Ltd, Tokyo)	1
RIBO-prep NA extraction kit (AmpliSense, Voisins-Le-Bretonneux)	1

### Toscana virus

**Figure 1:**
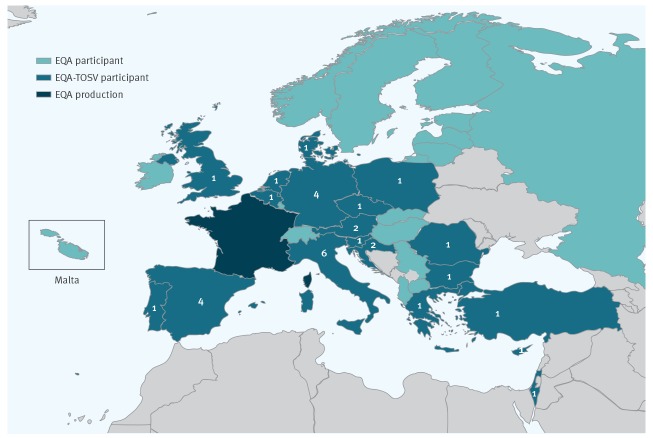
Number of laboratories per country that provided results for Toscana virus, external quality assessment for molecular detection of emerging neurotropic viruses, Europe (n = 32 laboratories)

EQA: external quality assessment; TOSV: Toscana virus.

Of the 51 laboratories, 32 laboratories in 19 countries (17 EU/EEA, one EU candidate, one other) tested the panel for the presence of TOSV RNA (Figure 1). Nineteen laboratories in 19 countries had no TOSV test available. Seven laboratories used a pan-phlebovirus RT-PCR only, 23 laboratories a TOSV-specific RT-PCR only and two laboratories used both type of tests in combination. Some laboratories used more than one TOSV-specific or pan-phlebovirus test (Table 2).

Blue: countries participating with TOSV RNA testing; dark blue: France (the Unite des Virus Emergents (National Reference Centre for arboviruses) at Aix Marseille University prepared and anonymised the panel); light blue: countries that submitted results for viruses other than TOSV.

**Table 2:** RT-PCR methods used for Toscana virus RNA detection, external quality assessment for molecular detection of emerging neurotropic viruses, Europe (n = 32 laboratories).

Target	Method	Number of laboratories	False-negative^a^
Toscana virus-specific			
TOSV N	Perez-Ruiz et al., 2007 [29]^b^	13	1 (lineage B)
TOSV N	Weidmann et al., 2008 [30]^b^	5	None
TOSV N	Brisbarre et al., 2015 [31]^b^	2	None
TOSV L	Sanchez-Seco et al., 2003 [43]^c^	1	1 (lineage B)
TOSV various	Own design^d^	5	1 (lineage B)
TOSV N	Progenie (commercial)^b^	1	None
Pan-phlebovirus			
Pan-phlebo L/N^e^	Sanchez-Seco et al., 2003 [43]	7	1 (lineage A), 2 (lineage B)
Pan-phlebo N	Lambert and Lanciotti, 2009 [44]	1	1 (lineage B)
Pan-phlebo unknown	Own design^d^	1	None

TOSV: Toscana virus.

^a^ The missed TOSV lineage is indicated between brackets.

^b^ Included in statistical analysis as classified as virus-specific real-time RT-PCR.

^C^ Included in statistical analysis as classified as conventional RT-PCR.

^d^ Excluded from statistical analysis as no distinction could be made whether it is real-time RT-PCR or conventional RT-PCR.

^e^ Not all participants indicated which of two pan-Phlebo RT-PCRs in the reference was used.

Excluding TOSV-specific assays for which no information was available (n = 10), TOSV-specific real-time tests (n = 42) provided false-negative results significantly less frequently than all other tests together (pan-phlebo, classic and nested RT-PCR; n = 20; p = 0.011). Thirty-one of the 32 laboratories detected TOSV RNA correctly in sample #1 (lineage A) and 28 laboratories detected TOSV RNA correctly in sample #2 (lineage B). The RT-PCR tests used by laboratories that missed the presence of TOSV in sample #1 (n = 1) or in sample #2 (n = 6) are presented in Table 2. One laboratory falsely detected TBEV RNA besides TOSV RNA in sample #1 (Table 3).

**Table 3:** Summary of results of laboratories in the external quality assesment on molecular diagnostics of emerging neurotropic viruses, Europe (n = 51)

Sample ID	1^a^	2	3	4	5	6	7	8	9	10
Virus	TOSV (lineage A)	TOSV (lineage B)	USUV	WNV (lineage 1)	WNV (lineage 2)	TBEV	Negative	Negative	Negative	Negative
Concentration	1.57 x 10^5^ RNA cp/0.4mL	1.24 x 10^5^ RNA cp/0.4mL	6.34 x 10^3^ RNA cp/0.4mL	7.2 x 10^4^ RNA cp/0.4mL	4.96 x 10^5^ RNA cp/0.4mL	5.06 x 10^4^ RNA cp/0.4mL	n/a	n/a	n/a	n/a
Total correct positive when tested for the specific virus	31/51	28/51	23/51	42/51	46/51	37/51	n/a	n/a	n/a	n/a
Total correct positive when not tested for the specific virus	17/51	18/51	19/51	2/51	2/51	8/51	n/a	n/a	n/a	n/a
**Total correct**	**48/51**	**46/51**	**42/51**	**44/51**	**48/51**	**45/51**	**47/51**	**48/51**	**49/51**	**48/51**
Total partially correct: identification at the genus level	0/51	0/51	1/51	2/51	1/51	2/51	n/a	n/a	n/a	n/a
False	4/51	5/51	5/51	5/51	2/51	4/51	**3/51**	**3/51**	**1/51**	**3/51**
**Total sentivity**	**31/32**	**28/32**	**21/28**	**42/48**	**46/48**	**36/42**	n/a	n/a	n/a	n/a

n/a: not applicable; TBEV: tick-borne encephalitis virus; TOSV: Toscana virus; USUV: Usutu virus; WNV: West Nile virus.

^a^ Number > 100% as one laboratory submitted both a correct result (positive for TOSV) and one false result (positive for TBEV) for this sample.

### West Nile virus

**Figure 2:**
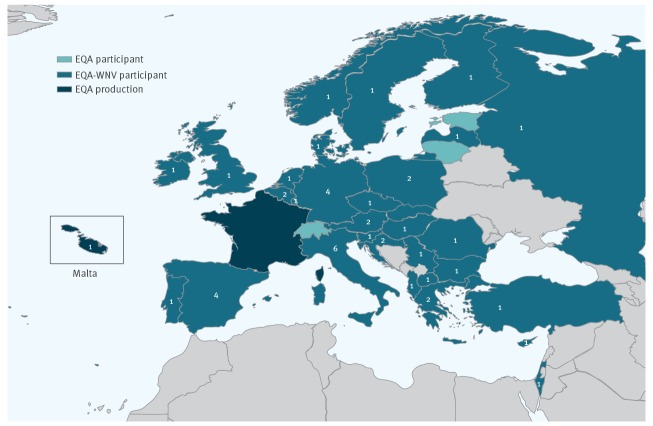
Number of laboratories per country that provided results for West Nile virus, external quality assessment for molecular detection of emerging neurotropic viruses, Europe (n = 48 laboratories)

EQA: external quality assessment; WNV: West Nile virus.

Forty-eight laboratories in 32 countries (26 EU/EEA, four EU candidates and two other) tested the panel for WNV RNA (Figure 2). One laboratory used a pan-flavi RT-PCR test while 28 laboratories used a WNV-specific RT-PCR. Eighteen laboratories used both a pan-flavi and WNV-specific RT-PCR, but the questionnaire did not allow linking the result with either assay. One laboratory did not report what type of test was used. Some laboratories used more than one RT-PCR test (Table 4). The diversity of WNV-specific tests used was high with a total of 25 different tests. Excluding WNV-specific assays for which no information was available (n = 4), there was no statistically significant difference between results provided by WNV-specific real-time tests (n = 29) and all other tests together (real-time pan-flavi, classic and nested RT-PCR; n = 22; p = 0.38).

Blue: countries participating with WNV RNA testing; dark blue: France (the Unite des Virus Emergents (National Reference Centre for arboviruses) at Aix Marseille University prepared and anonymised the panel); light blue: countries that submitted results for viruses other than WNV.

**Table 4:** RT-PCR methods used for West Nile virus RNA detection, external quality assessment for molecular detection of emerging neurotropic viruses, Europe (n = 48 laboratories)

Target	Method	Number of laboratories	False-negative^a^
West Nile virus-specific			
WNV 5'-UTR/C	Linke et al., 2007 [45]^b^	11	1 (lineage 2)
WNV NS2A	Eiden et al., 2010 [46]^b^	2	None
WNV 3'-UTR	Tang et al., 2006 [47]^b^	1	None
WNV 3’UTR	Lanciotti et al., 2000 [48]^b^	1	None
WNV E/NS1	Shi et al., 2001 [49]^b^	1	None
WNV NS3	Chaskopoulou et al., 2011 [50]	1	None
WNV various	Own design	3	1 (lineage 1)
WNV unknown	Altona RealStar (commercial)^b^	5	1 (lineage 2)
WNV unknown	Qiagen Artus (commercial)^b^	3	1 (lineage 1)
WNV unknown	Fast Track Tropical fever Core (commercial)^b^	3	1 (lineage 1)
WNV unknown	Sacace (commercial)^b^	1	None
WNV unknown	Amplisense (commercial)^b^	1	None
Pan-flavivirus			
Pan-flavi NS5	Scaramozzino et al., 2001 [51]	7	None
Pan-flavi NS5	Sanchez-Seco et al., 2005 [52]	3	None
Pan-flavi NS5	Moureau et al., 2007 [53]	2	1 (lineage 1)
Pan-flavi NS5	Patel et al., 2013 [54]	2	None
Pan-flavi NS5	Briese et al., 1999 [55]	1	None
Pan-flavi NS5	Vina-Rodriguez et al., 2017 [56]	1	None
Pan-flavi NS5	Vazques et al., 2012 [57]	1	None
Pan-flavi unknown	Own design	2	None
Pan-flavi unknown	Genekam (commercial)	1	None
Pan-flavi unknown	TibMolBiol (commercial)	1	None
Information not provided^c^		1	1 (lineage 1)

WNV: West Nile virus.

^a^ WNV lineage missed indicated between brackets.

^b^ Included in statistical analysis as classified as virus-specific real-time RT-PCR.

^c^ Excluded from statistical analysis since cannot be classified as real-time RT-PCR or conventional RT-PCR.

Forty-two of 48 laboratories that tested the panel for the presence of WNV RNA detected WNV RNA correctly in sample #4 (lineage 1), while one laboratory indicated to have detected flavivirus RNA. Five laboratories falsely scored sample #4 as negative. WNV RNA was correctly identified in sample #5 (lineage 2) by 46 of 48 laboratories. Of the two laboratories providing a false-negative result for sample #5, one laboratory reported the presence of TBEV RNA in sample #5; the other reported the presence of flavivirus RNA, although it did not claim to test for WNV (Table 3).

### Usutu virus

**Figure 3:**
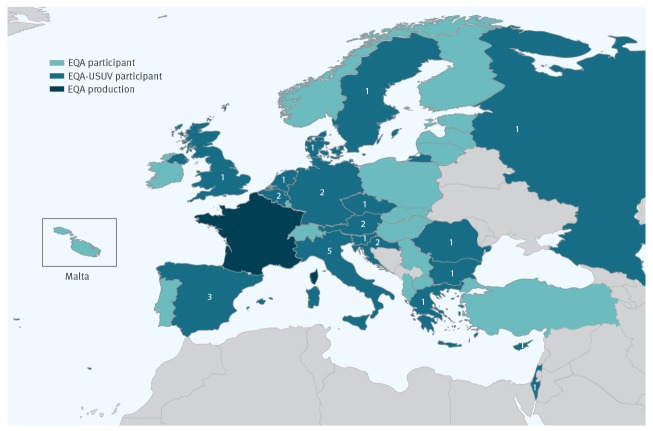
Number of laboratories per country that provided results for Usutu virus, external quality assessment for molecular detection of emerging neurotropic viruses, Europe (n = 28 laboratories)

EQA: external quality assessment; USUV: Usutu virus.

Twenty-eight laboratories in 18 countries (16 EU/EEA, two other) tested the panel for USUV (Figure 3). Six laboratories used a pan-flavivirus RT-PCR test only, 15 used an USUV-specific RT-PCR only and seven used both a pan-flavivirus and USUV-specific RT-PCR, but it was impossible to trace which one was used to provide the submitted results. Some laboratories used more than one USUV-specific or pan-flavivirus RT-PCR test. There was a lot of variation in USUV-specific RT-PCRs used, with 17 different test systems (Tables 3 and 5).

Blue: countries participating with USUV RNA testing; dark blue: France (the Unite des Virus Emergents (National Reference Centre for arboviruses) at Aix Marseille University prepared and anonymised the panel); light blue: countries that submitted results for viruses other than USUV.

**Table 5:** RT-PCR methods used for Usutu virus RNA detection, external quality assessment for molecular detection of emerging neurotropic viruses, Europe (n = 28 laboratories)

Target	Method	Number of laboratories	False-negative
Usutu virus-specific			
USUV NS5	Nikolay et al., 2014 [58]^a^	11	1
USUV NS5	Cavrini et al., 2011 [59]^a^	5	none
USUV NS1	Jöst et al., 2011 [60]^a^	2	none
USUV NS5	Weissenböck et al., 2013 [61]^a^	1	none
USUV 3'UTR	Del Amo et al., 2013 [62]^a^	1	none
USUV unknown	Own design^b^	4	none
Pan-flavivirus			
Pan-flavi NS5	Scaramozzino et al., 2001 [51]	5	3
Pan-flavi NS5	Sanchez-Seco et al., 2005 [52]	3	3
Pan-flavi NS5	Patel et al., 2013 [54]	2	none
Pan-flavi NS5	Vina-Rodriguez et al., 2017 [56]	1	1
Pan-flavi NS5	Vazques et al., 2012 [57]	1	1
Pan-flavi unknown	Own design	2	none
Pan-flavi unknown	Genekam (commercial)	1	none

USUV: Usutu virus.

^a^ Included in statistical analysis as classified as virus-specific real-time RT-PCR.

^b^ Excluded from statistical analysis since cannot be classified as real-time RT-PCR or conventional RT-PCR.

Excluding USUV-specific assays for which no information was available (n = 4), USUV-specific real-time tests (n = 20) provided false negative results significantly less frequently than all other tests together (real-time pan-flavi, classic and nested RT-PCR; n = 15; p = 0019). Seven of the 28 laboratories that tested the panel for USUV missed the positive sample #6. The RT-PCR tests used by these laboratories are indicated in Table 5.

### Tick-borne encephalitis virus

**Figure 4:**
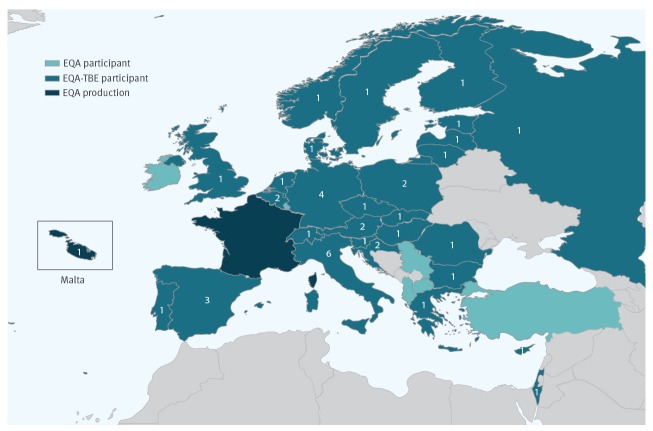
Number of laboratories per country that provided results for tick-borne encephalitis virus, external quality assessment for molecular detection of emerging neurotropic viruses, Europe (n = 42 laboratories)

EQA: external quality assessment; TBEV: tick-borne encephalitis virus.

Forty-two laboratories in 28 countries (25 EU/EEA, three other) tested the panel for TBEV RNA (Figure 4): seven used a pan-flavi RT-PCR test only, 24 used a TBEV-specific RT-PCR only and 11 used both a pan-flavi and TBEV-specific RT-PCR, however, the questionnaire did not permit to trace submitted results to one or the other assay. Moreover, some laboratories used more than one pan-flavivirus test. Table 6 gives an overview of the different RT-PCRs tests that were used on the EQA panel to detect TBEV RNA.

Blue: countries participating with TBEV RNA testing; dark blue: France (the Unite des Virus Emergents (National Reference Centre for arboviruses) at Aix Marseille University prepared and anonymised the panel); light blue: countries that submitted results for viruses other than TBEV.

**Table 6:** RT-PCR methods used for tick-borne encephalitis virus RNA detection, external quality assessment for molecular detection of emerging neurotropic viruses, Europe (n = 42 laboratories)

Target	Method	Number of laboratories	False-negative
Tick-borne encephalitis virus			
TBEV-3'-UTR	Schwaiger et al., 2003 [63]^a^	17	None
TBEV-NS1	Achazi et al., 2011 [64]^a^	5	None
TBEV-NS5	Puchhammer-Stöckl et al., 1995 [65]	2	None
TBEV-NS4	Bago et al., 2002 [66]^b^	1	None
TBEV-5'-UTR	Schrader et al., 1999 [67]	1	None
TBEV-E	Briggs et al., 2011 [68]	1	None
TBEV-E	Gäumann et al., 2010 [69]^a^	1	None
TBEV-5'-UTR	Klaus et al., 2010 [70]^a^	1	None
TBEV-3'-UTR	Brinkley et al., 2008 [71]^b^	1	None
TBEV-E	Andreassen et al., 2012 [72]^a^	1	None
TBEV-E	Skarpaas et al., 2006 [73]	1	None
TBEV-various	Own design^b^	2	None
TBEV Unknown	Genesig (commercial)^a^	1	1
TBEV Unknown	Amplisense (commercial)^a^	2	1
TBEV Unknown	Viasure (commercial)^a^	1	None
Pan-flavivirus			
Pan-flavi NS5	Scaramozzino et al., 2001 [51]	7	None
Pan-flavi NS5	Sanchez-Seco et al., 2005 [52]	3	2
Pan-flavi NS5	Moureau et al., 2007 [53]	1	None
Pan-flavi NS5	Patel et al., 2013 [54]	2	None
Pan-flavi NS5	Briese et al., 1999 [55]	1	None
Pan-flavi NS5	Vina-Rodriguez et al., 2017 [56]	1	None
Pan-flavi NS5	Vazques et al., 2012 [57]	1	None
Pan-flavi unknown	Own design	2	None
Pan-flavi unknown	Genekam (commercial)	1	None
Pan-flavi unknown	TibMolBiol (commercial)	1	None

TBEV: tick-borne encephalitis virus.

^a^ Included in statistical analysis as classified as virus-specific real-time RT-PCR.

^b^ Excluded from statistical analysis since cannot be classified as real-time RT-PCR or conventional RT-PCR.

Excluding TBEV-specific assays for which no information was available (n = 4), there was no statistically significant difference between results provided by TBEV-specific real-time tests (n = 29) and all other tests together (real-time pan-flavi, classic and nested RT-PCR n = 25; p = 1).

Thirty-six of 42 laboratories that tested the panel for the presence of TBEV RNA detected TBEV RNA correctly in sample #3. Four laboratories falsely scored sample #3 as negative including those using two commercial tests. One laboratory falsely indicated the presence of WNV RNA. One laboratory scored sample #3 as pan-flavi-positive only (Table 3).

### Contamination

Contamination issues were noticed in six of the 51 participating laboratories. Contamination issues involved detection of flavivirus, Zika virus, WNV or TBEV RNA in the negative control samples or in samples containing other specific viruses.

## Discussion

Fifty-one laboratories from 35 countries (28 EU/EEA, four EU pre-accession, three non-EU/EEA) participated in this EQA on molecular detection of emerging neurotropic viruses. Twenty-five laboratories in 16 countries (15 EU/EEA, one non-EU/EEA) reported capacity for testing of all four EQA target viruses. However, only 11 of the 25 scored the panel 100% correct. These 11 laboratories represented 10 EU/EEA countries and one non-EU/EEA country. Overall, the results of the EQA are not satisfactory. The capacity and capability for molecular detection needs to be improved in the vast majority of the participating laboratories because these four viruses demonstrate a growing burden on public health, have sympatric circulation (at least two of them) in several European countries and are indistinguishable clinically. It is important to underline that most of the participating laboratories were not first-line routine laboratories but national reference laboratories [3]. The fact that samples were missed by laboratories is of concern as the samples had RNA loads within the average of clinical relevance and were not intended to be at the detection limit to evaluate sensitivity. Another worrisome observation is the fact that six of 51 participating laboratories scored one or more of the viral RNA-negative samples positive, which is indicative of contamination issues and happened more frequently than in previous EQAs [21-23].

In our study, the total number of panels tested by each RT-PCR test did not allow statistically significant conclusions about specific methods that laboratories should be advised to use. Nevertheless, for TOSV and USUV, methods other than virus-specific real-time assays provided false-negative results more frequently than virus-specific real-time PCR tests. Although the same trend was not observed for WNV and TBEV, this could be taken into consideration by laboratories to improve the performance of their diagnostic capacity.

Because TOSV is endemic in countries surrounding the Mediterranean Sea, the majority of reference laboratories in Europe deal only with imported TOSV cases [24-28]. The neglected state of TOSV is reflected in the general absence of commercial tests, except for one which was used by one laboratory for the EQA panel. TOSV detection capacity had a geographical and laboratory coverage comparable to USUV, i.e. 32 laboratories in 19 countries which included all participating countries with known TOSV circulation (Croatia, Cyprus, France, Greece, Italy, Portugal and Spain). Bosnia and Herzegovina and Kosovo*, two other European countries with TOSV activity, did not participate in the EQA. Three TOSV lineages circulate in Europe, two of which were represented in the EQA panel, i.e. lineages A and B. The third lineage, lineage C, has only recently been discovered in Greece and Croatia and could not be included in the panel because the virus isolate was not available at the time. Of the 32 laboratories that tested for TOSV, four laboratories in four countries missed the TOSV lineage B sample; another laboratory in a fifth country missed the lineage A sample. At RT-PCR test level, lineage A was missed with one test while lineage B was missed six times by five RT-PCR tests of which four were conventional RT-PCR methods, despite the fact that the samples had similar viral loads. Apparently some laboratories used systems that were not sensitive enough for detection of TOSV lineage B strains, although this lineage is geographically most widely spread [4]. TOSV RNA loads provided in this EQA were in line with the virological findings in CSF [29-31,32,33]. The recent discovery of lineage C merits attention and the capacity of currently described assays to detect such strains need to be verified; since virological and genetic characterisation of this lineage is ongoing in Greece, inclusion of this lineage will be possible in future EQAs. At the country level, three of the five laboratories that missed a TOSV RNA-positive sample were located in a country endemic for TOSV. Better insight into the capability of TOSV molecular detection in Europe should be obtained with a dedicated EQA, including all three lineages at different viral loads, designed for a comparative evaluation of the RT-PCR methods described in the literature.

The widest geographical (32 countries) and laboratory (n = 48) coverage was for WNV testing. The WNV lineage 1 sample was missed by five laboratories in five EU/EEA countries that had never reported an autochthonous WNV case, while WNV lineage 2 was missed by two laboratories in two EU/EEA countries, one of which is endemic for WNV lineage 2. This was the third EQA of molecular detection of WNV within EVD-LabNet and its predecessor ENIVD [22,23]. The long history of WNV capability assessments and surveillance in Europe is likely to explain the good scores observed with WNV.

In this panel, USUV was the most recent emerging virus with still accumulating evidence of its relevance for public health and an increasing geographical distribution [13]. This might explain why the testing capability for USUV had the smallest geographical coverage (n = 18 countries) and number of laboratories (n = 28 laboratories). This was the first EQA that included USUV and there is no literature on clinically relevant viral loads in plasma. The concentration in this panel (1.6 × 10^4^ copies/mL) was in the range of detected viral loads for the closely related WNV in plasma [34-36]. Looking at the currently known geographical distribution of USUV in Europe, all countries with USUV circulation except Switzerland participated with USUV testing. The USUV-positive sample was missed by seven laboratories in four EU/EEA countries. To gain better insight in the robustness of USUV detection in Europe, a dedicated EQA including a concentration range of USUV genome copies in different matrices (whole blood, plasma and urine) is to be planned.

Although the geographical distribution of TBEV in Europe is broader than that of WNV and the total number of tick-borne encephalitis cases is higher, the number of laboratories participating with TBEV testing (n = 42) and their country coverage (n = 28) was smaller than for WNV. The TBEV sample was missed by four laboratories in three countries, of which two display endemic presence of TBEV. This was the second EQA including molecular detection of TBEV within EVD-LabNet and its predecessor ENIVD [21]. However, overall results could not be compared as our EQA only assessed TBEV testing based on one single RNA viral load.

Based on our results, we cannot give advice on what methods to use for the molecular detection of the four viruses. This requires assessment of the whole routine procedure from sample receipt to generation of a result. The performance in the EQA is a combination of the extraction method and the RT-PCR method used, as would routinely be the case when processing real-life diagnostic samples. The set-up of the current EQA cannot assess the influence of the extraction method or RT-PCR system on the final outcome per sample. The background data provided by the participants indicated an important diversity of the methods used for nucleic acid extraction (19 methods). It was impossible to link the extraction method to the quality of the results. To assess solely the quality of the RT-PCR, EQA panels consisting of extracted or synthetic RNA should be provided. Although our study was not designed to address the efficacy of the extraction technique per se, there are many arguments that favour automated extraction protocols over manual protocols. Automated extraction reduces the risk of cross-contamination, the turnaround and hands-on times, provide equivalent amounts of viral RNA and guarantee a better reproducibility compared with manual extraction [37–42]. EQA is an efficient tool to evaluate diagnostic procedures and to alert highlight where improvements are needed. Therefore, we recommend repeating the EQA for laboratories with unsatisfactory results, focusing at least on TOSV and USUV and investigating whether the required improvements are achieved. For these two viruses, we recommend real-time assays rather than classic or nested PCR protocols.

## Conclusion

Early detection of neurotropic arboviruses allows for timely risk assessment and risk management measures. We observed wide variation in both extraction methods and RT-PCR tests, showing a profound absence of standardisation across European laboratories. Overall, the results were not satisfactory and indicated a need for improvement of capacity and capability. Testing for WNV and TBEV, for which EQAs had been organised previously, showed better results than testing for USUV and TOSV for which this EQA was the first. This trend is important to consider and suggests that EQA exercises for TOSV and USUV should be repeated in order to assess whether successful improvements have been made.

## *Note

This designation is without prejudice to positions on status, and is in line with UNSCR 1244 and the ICJ Opinion on the Kosovo Declaration of Independence
